# What is the access to NHS fertility treatments for women with Mayer-Rokitansky-Küster-Hauser syndrome across England? A freedom of information study

**DOI:** 10.1136/bmjopen-2025-102664

**Published:** 2025-10-09

**Authors:** Kirsty Milford, Pedro Melo, Mehrunisha Suleman, Naomi Crouch, Isla Mair, Sarah Christine Armstrong

**Affiliations:** 1 Department of Gynaecology, University Hospitals Bristol and Weston NHS Foundation Trust, Bristol, UK; 2 University of Oxford Nuffield Department of Women’s and Reproductive Health, Oxford, UK; 3 University of Oxford Nuffield Department of Population Health, Oxford, UK; 4 Royal Navy, London, UK

**Keywords:** Reproductive medicine, Health Equity, Subfertility, Pregnancy

## Abstract

**Abstract:**

**Objectives:**

The aims of this study were (1) To investigate the availability of NHS funded in vitro fertilisation (IVF) treatment for individuals affected by Mayer-Rokitansky-Küster-Hauser syndrome (MRKH) from all Integrated Care Boards (ICBs) across England and (2) To assess the ethical implications of piecemeal funding for those with MRKH.

**Design:**

This was a mixed-methods study containing both quantitative and qualitative data. We filed freedom of information (FOI) act requests on 01/06/2023 for all 42 ICBs across England via secure email.

**Setting:**

The study focused on England.

**Participants:**

All 42 ICBs across England were contacted.

**Outcome measures:**

The FOI requests asked for information concerning the provision of funded IVF for uterine factor infertility, and if this included individuals with MRKH. Where assistance was available, we recorded what it comprised along the IVF cycle. If IVF was not offered, we recorded the rationale provided by the ICB.

**Results:**

Responses were received from all 42 ICBs across England. Seven stated that they would fund IVF and cryopreservation of embryos to women with MRKH and other absolute uterine factor infertility diagnoses (NHS Humber and North Yorkshire, NHS Dorset, NHS Devon, NHS Cornwall and Isles of Scilly, NHS Buckinghamshire, Oxford and Berkshire, NHS South Yorkshire and NHS West Yorkshire). However, the number of cycles, the length of cryopreservation and whether they would fund embryo transfer into a surrogate differed between ICBs.

Of the remainder, three (NHS Leicester, Leicestershire and Rutland, NHS Greater Manchester and NHS Hampshire and Isle of Wight) described some provision of fertility preservation (cryopreservation of oocytes or embryos) for women with uterine factor infertility, two of whom suggested their policy may include women with MRKH (NHS Greater Manchester and NHS Hampshire and Isle of Wight). Two ICBs (NHS Gloucester and NHS Bedford, Luton and Milton Keynes) explained that individual funding applications would be considered when made by clinicians on the patient’s behalf, but no information was provided on how many times requests had been made and granted. The remaining 30 ICBs explained that no part of a surrogacy pregnancy would be funded, owing to concerns around commercial surrogacy, which is illegal in the UK.

**Conclusions:**

This work has revealed that only a small proportion of ICBs (7/42, 17%) treat women with MRKH like any other woman applying for NHS fertility treatment. The study revealed that decisions by ICBs not to fund IVF treatments based on concerns about commercial surrogacy create significant inequities. It unfairly penalises individuals with MRKH who require surrogacy as part of their fertility treatment. These individuals face a unique set of reproductive challenges, and denying them access to NHS-funded IVF treatments exacerbates existing inequalities. Furthermore, if individuals with MRKH accept that the expenses of the surrogate will be met by them rather than the ICB, it is unjustifiable to deny them the IVF component of the treatment if they meet all the other criteria for eligibility. Moreover, the fact that some ICBs do fund IVF for individuals with MRKH indicates that legal concerns regarding surrogacy are unfounded and inconsistently applied. This discrepancy highlights the need for a standardised approach that ensures equitable access to fertility treatments across all regions.

**STRENGTHS AND LIMITATIONS OF THIS STUDY**

- A freedom of information study that covers all Integrated Care Boards (ICBs) in England to lend clarity on the status of funding for individuals with Mayer-Rokitansky-Küster-Hauser (MRKH) who desire a genetic child.
- Information on precisely what is funded along the in vitro fertilisation (IVF) cycle according to ICB.
- Rationale published for why funding is not offered for applicable ICBs.
- The study is limited to England. Scotland, Wales and Northern Ireland’s NHS were not approached due to their different health systems.
- The psychological impact of denying individuals with MRKH IVF has not been explored.

## Background

 Mayer-Rokitansky-Küster-Hauser (MRKH) syndrome is a rare congenital condition that affects the female reproductive tract (box 1). It occurs in at least 1:4500 female births.^1 2^ Although most cases are sporadic, some familial inheritance has been noted, and it appears to be an autosomal dominant trait with incomplete penetrance and variable expressivity.^2 3^ Characteristically, women with MRKH have a small or absent uterus, absent cervix and absent upper vagina, with normal external genitalia and secondary sexual characteristics.^4 5^ MRKH is a condition which sits under the umbrella term of a difference in sex development, and as such, care is offered in expert tertiary centres for complex congenital gynaecology. Multidisciplinary team care comprises paediatric and adolescent gynaecologists, psychologists, radiologists and geneticists.^6^ Those with MRKH typically have a karyotype of 46, XX. It is commonly not diagnosed until adolescence, often detected after investigation for primary amenorrhoea, and typically presents through gynaecology services. Treatment for the condition can broadly be considered as developing the vagina to allow comfortable penetrative intercourse and addressing fertility concerns. Both require significant psychological support, which is a mandatory element of care.^6^

Box 1 **Classification of Mayer-Rokitansky-Küster-Hauser**

- Mayer-Rokitansky-Küster-Hauser (MRKH 1), otherwise known as congenital absence of uterus and vagina and Müllerian aplasia, or Rokitansky syndrome. Characterised by aplasia of the uterus and the upper two-thirds of the vagina.
- MRKH 2, otherwise known as Müllerian duct aplasia, renal dysplasia and cervical somite anomalies syndrome, or genital renal ear syndrome. Characterised by additional differences such as anomalies in the structure of the fallopian tubes or kidneys, hearing difficulties, spinal deformities (most commonly scoliosis and sometimes vertebral fusion) and heart defects.

### Fertility options

Women with MRKH have uterine factor infertility, with no chance of spontaneous pregnancy; however, in most, ovarian function is preserved. The option to achieve a pregnancy using their own oocytes is currently restricted to in vitro fertilisation (IVF) with a surrogate to carry the pregnancy. Uterine transplantation to allow a woman with MRKH to carry a pregnancy may become an option in the future, but the procedure is in its infancy, with only one successful transplant having occurred in the UK to date.^7^ According to the International Society of Uterus Transplantation registry, a total of 45 uterine transplants were registered between 2012 and 2020 worldwide, of which the vast majority (n=44) were performed to treat recipients with MRKH (2). Successful transplantation was defined as regular spontaneous menses in the recipient.^3^ Those individuals receiving a uterus from a live donor had a 78% chance of achieving menstruation, but those receiving a uterus from a deceased donor had slightly lower menstruation rates at 64%.^3^ Uterine donation and transplantation are major operations with potential complications in both the donor (19%) and the recipient (18%). However, following a successful transplantation, approximately 80% of recipients achieved a pregnancy through IVF.^8^

Receiving a diagnosis of MRKH can have a profound psychological impact on those affected, with many studies reporting increased levels of anxiety and depression, feelings of incompleteness, doubts about female identity and low self-esteem.^9^ The diagnosis is typically made in the mid-adolescent years, which has the potential to disrupt education. In addition, individuals are likely to need vaginal treatment should they wish to develop the vaginal length for penetrative intercourse and may be embarking on vaginal dilation therapy in their later teenage years. These challenges can be compounded by the additional concern of infertility. Parenthood is often a crucial factor for those with MRKH, with a recent French survey showing that 88% of the 148 participants with MRKH expressed a desire for parenthood.^10^ 

Individuals with MRKH who wish to conceive using their own or donor oocytes need to navigate IVF as well as the often-complex process of finding a surrogate to be a gestational carrier for their embryo. Access to NHS-funded IVF treatment in England is determined by individual Integrated Care Boards (ICBs), which cover differing geographical areas. Each ICB sets their own criteria for funding fertility treatments, which can vary considerably.^11^ The funding criteria are often not transparent, with ICB policies not always published online for patients and healthcare staff to access. It is widely recognised that there is a significant disparity in the funding of IVF across ICBs in England, which is colloquially known as the postcode lottery.^12^ Those with MRKH often struggle to access NHS-funded IVF, even when they are committed to funding the surrogacy portion of the treatment themselves. This raises important ethical questions, particularly around equity and fairness, discrimination and autonomy.

The aims of this study were (1) To investigate the availability of NHS funded IVF treatment for individuals affected by MRKH from all ICBs across England and (2) To assess the ethical implications of piecemeal funding for those affected by MRKH.

## Methods

This was a mixed-methods study containing both quantitative and qualitative data recorded as the justification behind funding decisions. We filed freedom of information (FOI) act requests on 01/06/2023 for all 42 ICBs across England via email. The Freedom of Information Act 2000 is the provision under which the public can request information held by public authorities, including the NHS.^13^ The questions posed to ICBs are detailed in online supplemental appendix 1.

Responses were collated using a secure spreadsheet (Microsoft 365). Information recorded included whether health boards provided IVF for uterine factor infertility, and if this included individuals with MRKH. Where assistance was available, we recorded what it comprised. If IVF was not offered, we recorded the rationale provided by the ICB. Where necessary, individual ICBs were contacted again by a further FOI request to clarify to what degree fertility treatment was funded; for example, exploring how far through the IVF process the funding stretched, and whether any of the surrogacy costs would be met.

**Figure 1:**
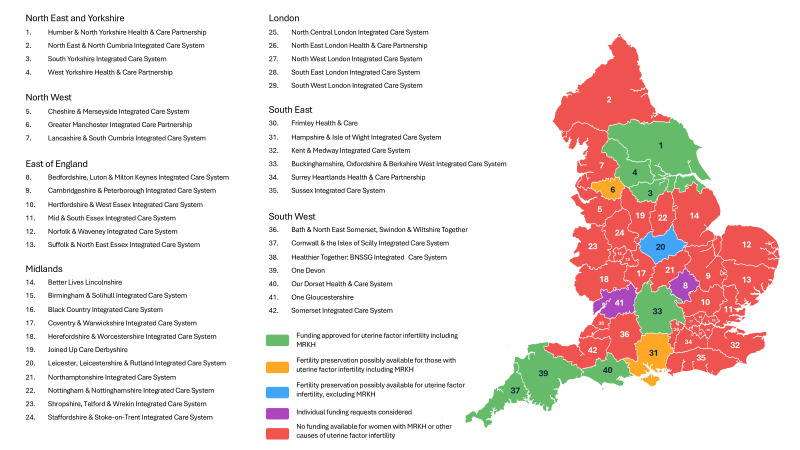
Integrated care board funding availability for women with MRKH seeking fertility treatment. MRKH, Mayer-Rokitansky-Küster-Hauser.

Figure 1 shows the availability of ICB funding for IVF for individuals affected by MRKH, with further details provided in table 1. Table 2 captures responses from ICBs where there is no immediately available funding for fertility treatment.

**Table 1:** Breakdown of the assisted reproductive technology available to women with Mayer-Rokitansky-Küster-Hauser

ICB	Funding availability for women with MRKH at the time of FOI request
NHS Humber and North Yorkshire	If standard ICB IVF eligibility criteria were met, for women with MRKH: One cycle of IVF is offered, with one further cycle offered if the first is abandoned for clinical reasons. Cryopreservation of embryos for 3 years or until 6 months following successful live birth. Embryo transfer into a surrogate is covered, which includes replacement of the resultant fresh embryos and subsequent sequential replacement of all frozen embryos derived from the cycle until pregnancy is successful, or all embryos have been exhausted. The costs associated with surrogacy are not covered.
NHS Dorset	If standard ICB IVF eligibility criteria were met, for women with MRKH: Fertility preservation, including creation of embryos, is offered to women with MRKH. Each case is assessed on an individual basis by the ICB. One cycle of IVF is offered, with a second under the following circumstances: if there is risk of hyperstimulation and the cycle is cancelled; if there is evidence of under-stimulation and a change in ovarian stimulation dose would be very likely to achieve stimulation; or if there is failed fertilisation and no embryos, whereupon a second cycle can be offered with or without donor sperm if clinically recommended. Oocyte or embryo cryopreservation up to 10 years, determined by individual case assessment. The costs associated with surrogacy are not covered. No provision for embryo transfer into a surrogate.
NHS Devon	If standard ICB IVF eligibility criteria were met, for women with MRKH: One cycle of IVF is offered, with a second under the following circumstances: when the cycle is abandoned for clinical reasons prior to oocyte collection; or there is failed fertilisation, or there are no suitable embryos to transfer. Cryopreservation of embryos for up to 5 years or implantation, whichever is sooner. The costs associated with surrogacy are not covered. No provision for embryo transfer into a surrogate.
NHS Cornwall and Isles of Scilly	If standard ICB IVF eligibility criteria were met, for women with MRKH: One cycle of IVF is offered, with a second under the following circumstances: when the cycle is abandoned for clinical reasons prior to oocyte collection; or there is failed fertilisation, or there are no suitable embryos to transfer. Cryopreservation of embryos or oocytes for up to 5 years or implantation, whichever is sooner. The costs associated with surrogacy are not covered. No provision for embryo transfer into a surrogate.
NHS Buckinghamshire, Oxford and Berkshire	If standard ICB IVF eligibility criteria were met, for women with MRKH: One cycle of IVF is offered. Cryopreservation of embryos for up to 3 years. The costs associated with surrogacy are not covered. No provision for embryo transfer into a surrogate.
NHS South Yorkshire	If standard ICB IVF eligibility criteria were met, for women with MRKH: One cycle of IVF is offered, with one further cycle offered if the first is abandoned for clinical reasons. Cryopreservation of embryos for 3 years or until 6 months following successful live birth. Embryo transfer into a surrogate is covered, which includes replacement of the resultant fresh embryos and subsequent sequential replacement of all frozen embryos derived from the cycle until pregnancy is successful, or all embryos have been exhausted. The costs associated with surrogacy are not covered.
NHS West Yorkshire	If standard ICB IVF eligibility criteria were met, for women with MRKH: One cycle of IVF is offered, with one further cycle offered if the first is abandoned for clinical reasons. Cryopreservation of embryos for 3 years or until 6 months following successful live birth. Embryo transfer into a surrogate is covered, which includes replacement of the resultant fresh embryos and subsequent sequential replacement of all frozen embryos derived from the cycle until pregnancy is successful, or all embryos have been exhausted. The costs associated with surrogacy are not covered.

FOI, freedom of information; ICB, integrated care board; IVF, in vitro fertilisation; MRKH, Mayer-Rokitansky-Küster-Hauser.

**Table 2:** Funding situation for those integrated care boards that do not offer assisted reproduction for uterine factor infertility or Mayer-Rokitansky-Küster-Hauser

Funding situation	ICB	FOI response
Fertility preservation possibly available for uterine factor infertility, including MRKH	NHS Greater Manchester	Fertility preservation (ie, freezing of oocytes or embryos) is available for women due to undergo cancer treatment, or individuals with a congenital condition which will affect fertility later in life. If surrogacy is required to achieve a pregnancy with these cryopreserved gametes, then this will not be funded by the ICB.
	NHS Hampshire and Isle of Wight	Fertility preservation (ie, freezing of oocytes or embryos) is available for women who are due to undergo a treatment that is likely to result in premature infertility (this may include, but is not limited to: surgery, chemotherapy, radiotherapy or endocrine treatment). If surrogacy is required to achieve a pregnancy with these cryopreserved gametes, then this will not be funded by the ICB.
Fertility preservation is available for uterine factor infertility, excluding MRKH	NHS Leicester, Leicestershire and Rutland	Fertility preservation (ie, freezing of oocytes or embryos) is available for women with cancer undergoing hysterectomy. Congenital reasons for the absence of the uterus, such as MRKH, are excluded.
No funding for women with MRKH or uterine factor infertility, but individual funding requests will be considered	NHS Gloucester	
	NHS Bedfordshire, Luton and Milton Keynes	
No funding available for women with MRKH or other causes of uterine factor infertility	NHS Stoke and Staffordshire	Will not fund any aspect of a surrogacy pregnancy.
	NHS Somerset	
	NHS Bristol, North Somerset and South Gloucestershire	
	NHS Surrey and Heartlands	
	NHS Hertfordshire and West Essex	
	NHS Coventry and Warwickshire	
	NHS Birmingham and Solihull	
	NHS North East London	
	NHS Lincolnshire	
	NHS Norfolk and Waverney	
	NHS Northamptonshire	
	NHS North East and Cumbria	
	NHS Shropshire, Telford and Wrekin	
	NHS SW London	
	NHS Suffolk and North East Essex	
	NHS Cheshire and Merseyside	
	NHS Black Country	
	NHS Derby and Derbyshire	
	NHS Nottingham and Nottinghamshire	
	NHS South East London	
	NHS North West London	
	NHS Lancashire and South Cumbria	
	NHS North Central London	
	NHS Cambridge and Peterborough	
	NHS Mid and South Essex	
	NHS Frimley	
	NHS Sussex	
	NHS Bath and North East Somerset	
	NHS Kent and Medway	Currently working on a funding plan to freeze all cycles, to allow those who can’t carry a pregnancy themselves to access treatment. Care regarding surrogacy would likely be privately funded. Current guidelines will not fund any aspect of a surrogacy pregnancy.
	NHS Herefordshire and Worcestershire	Stated N/A as fertility services for women without a womb are commissioned by NHS England, not the ICBs.*

* This is factually incorrect. Individual ICBs are responsible for determining funding.

FOI, freedom of information; ICB, integrated care board; MRKH, Mayer-Rokitansky-Küster-Hauser.

### Patient and public involvement

One of the authors (IM) has MRKH and has struggled to access NHS-funded fertility treatment. IM was involved at all stages of this project, from conceptualisation and design, through to editing of the manuscript. This ensured that the patient perspective was present throughout, and that the outcomes important to patients were central to our work.

## Results

Responses were received from all 42 ICBs across England. Seven stated that they provided fertility assistance to women with MRKH and other absolute uterine factor infertility diagnoses (table 1). Of the remainder, three (NHS Leicester, Leicestershire and Rutland, NHS Greater Manchester and NHS Hampshire and Isle of Wight) described some provision of fertility preservation (cryopreservation of oocytes or embryos) for women with uterine factor infertility, two of whom suggested their policy may include women with MRKH (table 2). Two ICBs (NHS Gloucester and NHS Bedford, Luton and Milton Keynes) explained that individual funding applications would be considered when made by clinicians on the patients’ behalf, but no information was provided on how many times requests had been made and granted (table 2).

The remaining 30 ICBs explained that no part of a surrogacy pregnancy would be funded, including IVF (table 2). Of these 30, one ICB (Kent and Medway) explained that they were updating their policy and were working on a funding plan for freeze-all embryo IVF cycles, to allow those who cannot carry a pregnancy themselves to access treatment. Further care regarding surrogacy would likely have to be privately funded (table 2).

Of the seven ICBs that funded some aspects of fertility treatment for individuals with MRKH, all stated that they did not fund any part of the surrogacy arrangement, with the reasoning that commercial surrogacy is illegal in the UK. All seven stated that they would fund IVF and cryopreservation of embryos (table 1). However, the number of cycles, length of cryopreservation and whether they would fund embryo transfer into a surrogate differed between ICBs.

## Discussion

To our knowledge, this paper represents the first FOI request project to explore the funding arrangement for fertility treatment for individuals with MRKH. It also highlights important ethical dilemmas posed by inequitable funding across England. Methodological limitations of this study include its focus on only England, as opposed to all countries of the UK. This decision was made owing to each Home Nation having a different health system, with England’s ICB system creating a unique challenge in terms of disparity in care. In addition, this study did not publish its protocol ahead of undertaking the study, but all outcomes that we set out to report have been reported. Finally, this study highlights the inequity of funded fertility treatment, but does not explore the psychological impact of this on women or their partners and family members. This is an area for further research.

Individuals with MRKH are born without a uterus, something entirely outside of their control, and an absolute cause of infertility. This work has revealed that only a small proportion of ICBs (7/42, 17%) treat these women like any other woman applying for fertility treatment through ICB channels. The remainder at the time of the FOI request did not fund any aspect of treatment (30/42, 71%) or required individual funding request applications that would be decided on a case-by-case basis (2/42, 5%). Two ICBs were equivocal, stating that fertility preservation may possibly be available (2/42, 5%), and one ICB explained that fertility preservation was only available for those with the absence of a uterus owing to reasons other than congenital causes (1/42, 2%).

All ICBs confirmed that they would not be involved in commercial surrogacy, owing to it being illegal in the UK. The main legislation surrounding surrogacy in the UK is the Surrogacy Act 1985 and the Human Fertilisation and Embryology Act 2008.^14 15^ The Law Commission defines surrogacy as when a woman bears a child on behalf of another couple or individual, who intend to become the child’s legal parents.^16^ Surrogacy is an altruistic act within the UK, and surrogates cannot receive payment for carrying a pregnancy; however, intended parents are responsible for reimbursing any reasonable expenses, which can include items like maternity clothes, travel expenses and loss of earnings. Typically, expenses can run into the tens of thousands of pounds. This is on top of the cost of fertility treatment: IVF with the potential need for intracytoplasmic sperm injection, where clinically indicated (eg, for concurrent male-factor infertility). This treatment adds between £5 000 and £12 000 to the cost of surrogacy, with the requirement for additional payments for frozen embryo transfers where treatment has previously been unsuccessful.^17^

The the most common reason (57%, n=24/42) given by ICBs for not funding fertility treatment for women with MRKH and other uterine factor infertility was that they would not be involved because of legal issues with commercial surrogacy. This is surprising, given that IVF treatment is only available through licensed clinics in the UK, which are tightly bound by the Human Fertilisation and Embryology Act 2008. Therefore, commercial surrogacy arrangements could never be performed according to present UK law. This suggests a profound misunderstanding regarding surrogacy and the role that assisted reproduction plays in it.

The data show that the decision by ICBs not to fund IVF treatments based on concerns about commercial surrogacy creates significant inequities. It unfairly penalises individuals with MRKH who require surrogacy as part of their fertility treatment. These individuals face a unique set of reproductive challenges, and denying them access to NHS-funded IVF treatments exacerbates existing inequalities. Furthermore, if individuals with MRKH accept that the expenses of the surrogate will be met by them rather than the ICB, it is unjustifiable to deny them the IVF component of the treatment, for which they would qualify under existing guidelines. Moreover, the fact that some ICBs do fund IVF for individuals with MRKH indicates that legal concerns regarding surrogacy are unfounded and inconsistently applied. This discrepancy highlights the need for a standardised approach that ensures equitable access to fertility treatments across all regions.

This inconsistency in funding reflects a broader pattern of unfairness within the NHS fertility treatment allocation system. The criteria for receiving NHS-funded IVF are not only geographically inconsistent but also often lack transparency, making it difficult for patients and healthcare providers to navigate the system. Factors such as age, previous children and relationship status can influence eligibility, leading to disparities even among those with similar medical conditions. While the NHS aims to provide equitable healthcare, the current system for allocating fertility treatment funding often fails to reflect this goal. Instead, it prioritises certain demographics over others without clear justification, inadvertently discriminating against those with rare conditions like MRKH who have specific and high-cost fertility needs.

Additional ethical concerns include addressing discrimination and stigma against individuals with MRKH, ensuring that policies do not perpetuate biases, and providing psychological support to those denied fertility treatments. Respecting patient autonomy requires that individuals with MRKH have access to comprehensive information and counselling to make informed decisions about their reproductive health.

Uterine transplant is a potential future treatment for those with MRKH to allow them to carry a pregnancy. However, it is not currently readily available outside of a research setting and carries significant morbidity for both donor and recipient. It is not currently funded by the NHS. A typical hysterectomy operation for the donor will take 10–12 hours, for example. The overall cost is likely to escalate beyond the cost of surrogacy, given it requires several operations, anti-rejection medication, at least one cycle of IVF, prolonged monitoring of recipients for at least a year after transplant and during pregnancy.^18^

People with MRKH often must explain their condition, and this was highlighted by the misunderstanding from three ICBs (NHS Lincolnshire, NHS Norfolk and Waveney and NHS Northamptonshire), who initially provided information on the help available to transgender people. This seems surprising for a condition which affects 1:4500 girls and highlights a general lack of understanding of the condition. These ICBs have excellent provision for cryopreservation of gametes for fertility preservation in transgender individuals; however, they do not extend the same provision to individuals with MRKH. This creates further barriers to healthcare through a lack of understanding.

The ethical implications of how resources are allocated to fertility treatment within the NHS must be carefully examined. It is important to assess the resource allocation and whether those with MRKH are adequately prioritised. A thorough cost-benefit analysis should be considered, and the unique needs of those with MRKH, ensuring financial considerations do not unjustly restrict access to necessary treatments. At the heart of this study lies the profound inequity that the individual ICB determined criteria for IVF creates. It is worth mentioning that those with MRKH are just one group of people who experience this inequity. Parallels can be drawn with those who experience tubal factor infertility, and the varying levels of fertility care (IVF or fertility preservation owing to increased risk of premature ovarian insufficiency) available to them.

MRKH often represents an unexpected and devastating diagnosis for individuals and their families. The significant distress which is often experienced is compounded by a lack of access to fertility services and confusion and misunderstanding about the condition. We are calling for there to be equal provision for access to fertility treatment across the ICBs and to improve education to reduce confusion about MRKH.

## Supplementary material

10.1136/bmjopen-2025-102664 online supplemental appendix 1

## Data Availability

Data are available upon reasonable request.

## References

[1] Herlin M, Bjørn A-MB, Rasmussen M (2016). Prevalence and patient characteristics of Mayer-Rokitansky-Küster-Hauser syndrome: a nationwide registry-based study. Hum Reprod.

[2] Kyei-Barffour I, Margetts M, Vash-Margita A (2021). The Embryological Landscape of Mayer-Rokitansky-Kuster-Hauser Syndrome: Genetics and Environmental Factors. Yale J Biol Med.

[3] Brännström M, Belfort MA, Ayoubi JM (2021). Uterus transplantation worldwide: clinical activities and outcomes. Curr Opin Organ Transplant.

[4] Troiano RN, McCarthy SM (2004). Mullerian duct anomalies: imaging and clinical issues. Radiology.

[5] Guerrier D, Mouchel T, Pasquier L (2006). The Mayer-Rokitansky-Küster-Hauser syndrome (congenital absence of uterus and vagina)--phenotypic manifestations and genetic approaches. J Negat Results Biomed.

[6] NHS (2019). Congenital gynaecological anomalies service specification. https://www.england.nhs.uk/publication/complex-gynaecology-congenital-gynaecological-anomalies-children-of-13-years-and-above-and-adults/.

[7] (2025). Womb transplant uk. https://wombtransplantuk.org/.

[8] Brännström M, Tullius SG, Brucker S (2023). Registry of the International Society of Uterus Transplantation: First Report. Transplantation.

[9] Bean EJ, Mazur T, Robinson AD (2009). Mayer-Rokitansky-Küster-Hauser syndrome: sexuality, psychological effects, and quality of life. J Pediatr Adolesc Gynecol.

[10] Sousa C, Carton I, Jaillard S (2023). Mayer-Rokitansky-Küster-Hauser syndrome patients’ interest, expectations and demands concerning uterus transplantation. J Gynecol Obstet Hum Reprod.

[11] Dept of Health and Social Care (2024). NHS-funded in vitro fertilisation (ivf) in england. https://www.gov.uk/government/publications/nhs-funded-ivf-in-england/nhs-funded-in-vitro-fertilisation-ivf-in-england.

[12] Tippett A (2023). Reproductive rights where conditions apply: an analysis of discriminatory practice in funding criteria against would-be parents seeking funded fertility treatment in England. Hum Fertil (Camb).

[13] legislation.gov.uk (2000). Freedom of information act 2000. https://www.legislation.gov.uk/ukpga/2000/36/contents.

[14] legislation.gov.uk (1985). Surrogacy arrangements act 1985. https://www.legislation.gov.uk/ukpga/1985/49/contents.

[15] legislation.gov.uk (2008). Human fertilisation and embryology act 2008. https://www.legislation.gov.uk/ukpga/2008/22/contents.

[16] Law Commission (2023). Building families through surrogacy: a new law. https://lawcom.gov.uk/project/surrogacy/#related.

[17] Human fertilisation and embryology authority In vitro fertilisation (ivf). https://www.hfea.gov.uk/treatments/explore-all-treatments/in-vitro-fertilisation-ivf/.

[18] Wilkinson S, Williams NJ (2016). Should uterus transplants be publicly funded?. J Med Ethics.

