# Supplementary material for: What is the access to NHS fertility treatments for women with Mayer-Rokitansky-Küster-Hauser syndrome across England? A freedom of information study
Source: BMJ Open. 2025 Oct 9;15(10):e102664. doi: 10.1136/bmjopen-2025-102664 (PMC12517017; doi:10.1136/bmjopen-2025-102664)
Supplement: online supplemental appendix 1 [file bmjopen-15-10-s001.docx]

1. Does your ICB fund IVF for women who do not possess a uterus, but meet all other set-out criteria, and are willing to fund the cost of a surrogate?
2. Does your ICB fund IVF for women with MRKH, but meet all other set-out criteria, and are willing to fund the cost of a surrogate?
