# Supplementary material for: What is the access to NHS fertility treatments for women with Mayer-Rokitansky-Küster-Hauser syndrome across England? A freedom of information study
Source: BMJ Open. 2025 Oct 9;15(10):e102664. doi: 10.1136/bmjopen-2025-102664 (PMC12517017; doi:10.1136/bmjopen-2025-102664)
Supplement: Supplementary file 2 [file bmjopen-2025-102664.reviewer_comments.pdf]

PEER REVIEW HISTORY

BMJ Open publishes all reviews undertaken for accepted manuscripts. Reviewers are asked to complete a checklist review form and are provided with free text boxes to elaborate on their assessment. These free text comments are reproduced below.

ARTICLE DETAILS

Title (Provisional)

What is the access to NHS fertility treatments for women with Mayer-Rokitansky-Küster-Hauser Syndrome across England? A freedom of information study.

Authors

Milford, Kirsty; Melo, Pedro; Suleman, Mehrunisha; Crouch, Naomi; Mair, Isla; Armstrong, Sarah Christine

VERSION 1 - REVIEW

**Reviewer**	**1**
**Name**	**Dewi, Ayu Putu Madri**
**Affiliation**	**Amsterdam UMC Location AMC, Epidemiology and Data Science**
**Date**	**08-Apr-2025**
**COI**	**None**

Manuscript Title: Inequity of access to NHS fertility treatments for women born without a uterus (Mayer-Rokitansky-Küster-Hauser (MRKH) Syndrome) in England

I am an Open Science researcher, who has checked your manuscript for its adherence to a number of Open Science practices.

Below you find the checklist that I used to assess your manuscript for adherence to a number of these practices.

Based on this assessment, I would suggest the following refinements/additions:

1. Availability of a protocol: Please provide a (link to the) the protocol.

2. Pre-registration: Please provide information on whether and where the study was pre-registered.

3. Open Materials: OK

4. Open Data: Please provide a statement about whether and where the data used for this study can be accessed

5. Open Code: Please provide (a link to) the code used for the analyses in this study.

6. Citations: Please provide citations for your materials/data/codes.

7. Software and tools used are described: Please explicitly mention which software and tools were used in your study, including references to versions.

8. Open access publication: OK

9. Reporting checklist provided: OK

10. Preprint: Please state explicitly whether and where the preprint of this study can be found.

**Reviewer**	**2**
**Name**	**Yasmin, Ephia**
**Affiliation**	**University College London Hospitals**
**Date**	**25-Jun-2025**
**COI**	**None**

The survey addresses an important concern about uterine factor infertility and parity of access to funding. It highlights disparity of access.

The study accesses only 42 ICBs. I would recommend accessing the policy of all ICBs as they are in the public domain. I would also suggest that they reach out to NI, Scotland and Wales. The survey will be best served by including the 4 nations.

The study should also mention if absolute tubal factor should also be included. As MRKH is not associated with a risk of premature ovarian insufficiency, how egg or embryo freezing in this context is clinically sound. Should we then freeze eggs or embryos when women lose both fallopian tubes to disease or ectopic pregnancy? If outside the remit of the survey, they need to make this clear as the rationale presented for MRKH can be easily extended to other scenarios.

**Reviewer**	**3**
**Name**	**Vash-Margita, Alla**
**Affiliation**	**Yale University School of Public Health, Obstetrics, Gynecology and Reproductive Sciences**
**Date**	**18-Jul-2025**
**COI**	**None**

This is an important topic that is understudied at present time. I commented the authors for dedicating the time and effort in conducting this research.

I have minor comments:

Page 2, line 51 - add ref Kyei-Barffour I et al. The Embryological Landscape of Mayer-Rokitansky-Kuster-Hauser Syndrome: Genetics and Environmental Factors. Yale J Biol Med. 2021 to describe genes that have been implicated in MRKH.

Page 3, line 21 - add scoliosis for most common vertebral anomalies

Please, discuss limitation of the study. For example, authors state that there is a need to provide psychological support to those denied fertility treatments (page 11, line 16-17), but there is no evidence presented in this specific population about impact of denied or unaccessible fertility treatment. This should be specified under limitations.

VERSION 1 - AUTHOR RESPONSE

Reviewer 1
1) Availability of a protocol: Please provide a (link to the) the protocol. We regret that no protocol was published prospectively.
2) Pre-registration: Please provide information on whether and where the study was pre-registered.
I'm sorry to report that the study was not pre-registered, but I have now registered it with University Hospitals Bristol and Weston NHS Foundation Trust.
4) Open Data: Please provide a statement about whether and where the data used for this study can be accessed.
The following has been placed into the manuscript: Data for this study can be requested by emailing the senior author: s.armstrong2@nhs.net
5. Open Code: Please provide (a link to) the code used for the analyses in this study.
Not applicable - no analyses were undertaken.
6. Citations: Please provide citations for your materials/data/codes.
Not applicable.
7. Software and tools used are described: Please explicitly mention which software and tools were used in your study, including references to versions.
One software tool was used - Microsoft Excel. I have added in brackets 'Microsoft 365' after the text 'responses were collated using a secure spreadsheet'. I hope this is ok.
10. Preprint: Please state explicitly whether and where the preprint of this study can be found.
There is is no preprint of this study.

Reviewer 2

1) The study accesses only 42 ICBs. I would recommend accessing the policy of all ICBs as they are in the public domain. I would also suggest that they reach out to NI, Scotland and Wales. The survey will be best served by including the 4 nations.

Thank you Ephia for your thoughtful comment. We agree that one paper with information from all Home Nations would be ideal, but as an authorship team we consciously chose to just focus on England for this particular study. The reason for this is that each Home Nation has a unique health system. ICBs are unique to England, with Scotland having Health and Social Care Integration Partnerships, Wales having Local Health Boards, and Northern Ireland having Heath and Social Care Organisations, which lends significant complexity in gathering information expediently. As a group, we plan to publish findings from these Home Nations, but in order to expedite dissemination of up to date and relevant information

regarding England, we request that these papers can be published separately (this one, and a subsequent Home Nation one).

2) The study should also mention if absolute tubal factor should also be included. As MRKH is not associated with a risk of premature ovarian insufficiency, how egg or embryo freezing in this context is clinically sound. Should we then freeze eggs or embryos when women lose both fallopian tubes to disease or ectopic pregnancy? If outside the remit of the survey, they need to make this clear as the rationale presented for MRKH can be easily extended to other scenarios.

Thank you for highlighting the parallels of absolutely infertility for those with MRKH and those with tubal factor infertility and how funding decisions should be harmonious across ICBs for women who lose function of both tubes. I have added the following paragraph into the discussion which I hope addresses your point:

At the heart of this study lies the profound inequity that individual ICB determined criteria for IVF creates. It is worth mentioning that those with MRKH are just one group of people who experience this inequity. Parallels can be drawn with those who experience tubal factor infertility, and the varying levels of fertility care (IVF or fertility preservation owing to increased risk of premature ovarian insufficiency) available to them.

Reviewer 3:
1) Page 2, line 51 - add ref Kyei-Barffour I et al - reference added.

2) Page 3, line 21 - add scoliosis for most common vertebral anomalies - done

3) Please, discuss limitation of the study. For example, authors state that there is a need to provide psychological support to those denied fertility treatments (page 11, line 16-17), but there is no evidence presented in this specific population about impact of denied or unaccessible fertility treatment. This should be specified under limitations.

Thank you for this comment. This limitation has now been acknowledged in the 'strengths and limitations of this study' as well as in the discussion. Please see tracked changes.
